# Neural responses to naturalistic audiovisual speech are related to listening demand in cochlear implant users

**DOI:** 10.3389/fnhum.2022.1043499

**Published:** 2022-11-07

**Authors:** Bowen Xiu, Brandon T. Paul, Joseph M. Chen, Trung N. Le, Vincent Y. Lin, Andrew Dimitrijevic

**Affiliations:** ^1^Evaluative Clinical Sciences Platform, Sunnybrook Research Institute, Toronto, ON, Canada; ^2^Department of Psychology, Toronto Metropolitan University, Toronto, ON, Canada; ^3^Department of Otolaryngology – Head and Neck Surgery, Sunnybrook Health Sciences Centre, Toronto, ON, Canada; ^4^Department of Otolaryngology – Head and Neck Surgery, Faculty of Medicine, University of Toronto, Toronto, ON, Canada

**Keywords:** speech tracking, EEG, listening in noise, attention, temporal response function, movies, naturalistic stimuli, cochlear implant

## Abstract

There is a weak relationship between clinical and self-reported speech perception outcomes in cochlear implant (CI) listeners. Such poor correspondence may be due to differences in clinical and “real-world” listening environments and stimuli. Speech in the real world is often accompanied by visual cues, background environmental noise, and is generally in a conversational context, all factors that could affect listening demand. Thus, our objectives were to determine if brain responses to naturalistic speech could index speech perception and listening demand in CI users. Accordingly, we recorded high-density electroencephalogram (EEG) while CI users listened/watched a naturalistic stimulus (i.e., the television show, “The Office”). We used continuous EEG to quantify “speech neural tracking” (i.e., TRFs, temporal response functions) to the show’s soundtrack and 8–12 Hz (alpha) brain rhythms commonly related to listening effort. Background noise at three different signal-to-noise ratios (SNRs), +5, +10, and +15 dB were presented to vary the difficulty of following the television show, mimicking a natural noisy environment. The task also included an audio-only (no video) condition. After each condition, participants subjectively rated listening demand and the degree of words and conversations they felt they understood. Fifteen CI users reported progressively higher degrees of listening demand and less words and conversation with increasing background noise. Listening demand and conversation understanding in the audio-only condition was comparable to that of the highest noise condition (+5 dB). Increasing background noise affected speech neural tracking at a group level, in addition to eliciting strong individual differences. Mixed effect modeling showed that listening demand and conversation understanding were correlated to early cortical speech tracking, such that high demand and low conversation understanding occurred with lower amplitude TRFs. In the high noise condition, greater listening demand was negatively correlated to parietal alpha power, where higher demand was related to lower alpha power. No significant correlations were observed between TRF/alpha and clinical speech perception scores. These results are similar to previous findings showing little relationship between clinical speech perception and quality-of-life in CI users. However, physiological responses to complex natural speech may provide an objective measure of aspects of quality-of-life measures like self-perceived listening demand.

## Introduction

Cochlear implants (CI) can successfully restore hearing for many individuals with profound-to-severe sensorineural hearing loss. Despite increases in post-implantation speech perception scores and quality-of-life (QoL) for most CI users (e.g., Dillon et al., 2018; Fabie et al., 2018), not all individuals achieve a favorable level of speech performance or QoL (e.g., Holden et al., 2013; Capretta and Moberly, 2016). Studies examining post-implantation outcomes have focused on etiology of hearing loss, duration of deafness, age at implantation, the amount of residual hearing, and device differences (e.g., Blamey et al., 2012; Lazard et al., 2012; James et al., 2019; Kurz et al., 2019). However, these factors only account for 10–22% of the outcome variability (Blamey et al., 2012; Lazard et al., 2012). Furthermore, the correlation between clinical speech perception test and subjective QoL questionnaire outcomes appears to be weak to moderate at best, with some studies reporting no significant relationship (e.g., Ramakers et al., 2017; McRackan et al., 2018; Thompson et al., 2020). Accordingly, QoL does not necessarily improve after cochlear implantation even if a desired level of speech understanding is reached. These observations motivated the development of clinical testing procedures that are sensitive to post-implant changes to QoL.

Clinical speech tests may fail to reliably predict QoL changes because current clinical testing materials do not fully capture aspects of real-world listening. Clinical tests take place with participants centered in a sound-attenuated booth with minimal reverberation where speech and background noise stimuli are presented at fixed, equidistant positions around the azimuth (Dunn et al., 2010; Minimum Speech Test Battery (MSTB) For Adult Cochlear Implant Users, 2011; Spahr et al., 2012). Additionally, the properties of speech and background noise stimuli themselves are often constant in terms of loudness and content. In contrast, listening in everyday life often occurs in dynamic complex environments where speech is often accompanied by fluctuating background noise, competing speech sources, and with varying access to visual (speech) input that influences speech comprehension. CI users often report that visual articulatory cues (i.e., lip, tongue, and teeth movements that accompany speech) are missing from clinical testing (Dorman et al., 2016). Visual articulatory cues are correlated with properties of the auditory speech signal and provide information that assists CI users in interpreting ambiguous speech, as demonstrated through the McGurk effect (Rouger et al., 2008; Stropahl et al., 2017). Results from neuroimaging studies suggest that visual articulatory cues increase selective attention, speech intelligibility, and neural speech tracking (the entrainment of the neural response to the attended speech stimuli) compared to audio-only speech-in-noise tasks, especially in individuals with hearing loss (Zion Golumbic et al., 2013; O’Sullivan et al., 2019; Puschmann et al., 2019). On the other hand, background noise and reverberation negatively affects word recognition and listening effort (Picou et al., 2016). The quality of neural speech tracking has also been shown to decrease in the presence of noise or competing sound sources; background noise has been demonstrated to attenuate the brain response around 100 ms after a change in the speech envelope amplitude (Ding and Simon, 2012; Fuglsang et al., 2017; Petersen et al., 2017).

In these instances, the burden of degraded speech and environmental distractions can increase the overall cognitive demand required to adequately comprehend speech (Rönnberg et al., 2010; Picou et al., 2016). Listening to speech in noise can impose a strong demand on cognitive resources due to increased engagement of working memory (Rönnberg et al., 2010, 2013) and attention processes (Shinn-Cunningham and Best, 2008). In complex listening environments where the sound quality of speech is reduced, listeners must apportion their limited cognitive resources toward attending to the targeted speech source, in addition to processing and storing auditory information (Rönnberg et al., 2013; Pichora-Fuller et al., 2016; Peelle, 2018). This is especially concerning for individuals with hearing loss who on average report significantly increased listening effort and fatigue as scored through subjective measures and indirect measures of brain activity (McGarrigle et al., 2014). Along with the innate loss of spectral resolution due to the CI processor, the demand of comprehending degraded speech places additional strain on the cognitive resources of CI users and can lead to decreased motivation and effort in engaging in social situations (Pichora-Fuller et al., 2016; Castellanos et al., 2018). Perceived cognitive demand and listening effort has been found to be a primary factor that is detrimental to QoL amongst individuals with hearing loss, especially in CI users (Hughes et al., 2018; Holman et al., 2019).

Current understanding of neural processes underlying the management of cognitive resources when listening in complex environments is limited. Including neural speech tracking, various methods have been devised to measure cognitive demand at a neural level. Alpha power (power of neural oscillations between 8 and 12 Hz) as a potential neural marker of effort and demand has been studied extensively (Foxe and Snyder, 2011; Klimesch, 2012; Obleser et al., 2012; Strauß et al., 2014; Wostmann et al., 2015; Petersen et al., 2015; Dimitrijevic et al., 2019; Magosso et al., 2019; Price et al., 2019; Hauswald et al., 2020; Hjortkjær et al., 2020; Paul et al., 2021). Increased alpha power in adverse listening conditions is hypothesized to represent the suppression of neural responses in brain regions unrelated to the task at hand (Payne and Sekuler, 2014; Strauß et al., 2014; Wilsch et al., 2015) and may reflect a conscious effort to ignore task-irrelevant stimuli (Petersen et al., 2015). Indeed, an increase in parietal alpha power has been observed during working memory tasks in individuals with hearing loss when both background noise and task difficulty increases (Petersen et al., 2015; Paul et al., 2021). A positive relationship between listening effort and alpha power in the left inferior frontal gyrus (IFG) was also observed in CI users performing a digits speech-in-noise task, suggesting that language networks are involved in self-perceived listening effort (Dimitrijevic et al., 2019). When combined with visual stimuli, an increase in alpha power is seen in the parieto-occipital region when individuals attend to audio that is incongruent to the presented visual cues, suggesting a suppression of the unrelated incoming visual stimuli (O’Sullivan et al., 2019). Therefore, alpha power potentially reflects a gating mechanism toward incoming sensory stimuli in a manner relevant to task goals and may serve as a marker of cognitive demand during listening in noisy environments.

While previous literature links alpha power and speech tracking to cognitive demand, demand appears to affect speech tracking, cognitive engagement, and alpha power differently, with speech envelope coherence demonstrating an inverted-U pattern while alpha power declines overall (Hauswald et al., 2020). Furthermore, it is unclear whether neural speech tracking and alpha power reflect self-perceived cognitive demand compared to current clinical speech perception measures. Therefore, the current objectives of the study are to first measure neural speech tracking in CI users using real-world speech, and then evaluate the effects of increasing background noise and the addition of visual cues on the quality of neural speech tracking. Then, we will quantify the relationship of neural speech tracking to the subjective self-reported level of cognitive demand, self-perceived word understanding, and self-perceived conversation understanding. Self-perceived word understanding and conversation understanding will be measured separately in the planned analyses, since the number of perceived words may not always provide enough context to understand a conversation. Based on previous research regarding effort-related alpha oscillations, we conducted a secondary analysis of alpha band power over the superior-parietal and left IFG regions. We hypothesize that:

1. Self-rated cognitive demand during speech listening will decrease with the presence of visual cues but increase in response to background noise levels.
2. The degree of speech tracking will be reduced by increasing background noise levels and enhanced by the presence of visual cues. Similarly, the presence of visual cues will result in a decrease in alpha power.
3. Neural speech tracking and alpha power will be related to subjective self-reports of cognitive/mental demands and words/conversation understanding.

## Materials and methods

### Participants

Participant inclusion criteria consisted of adult CI users between the ages of 18–80 that have native or bilingual fluency in English, with at least 1 year of experience with their implants. As the current objective is to quantify the relationship between clinical speech perception tests and self-perceived cognitive demand in a general CI user population, potential participants were not excluded based on etiology of hearing loss, age of hearing loss onset, performance on clinical speech perception tests, or sidedness of implantation (Table 1). Eighteen adult CI users were recruited from Sunnybrook Health Sciences Centre Department of Otolaryngology. Three participants were excluded due to poor electroencephalogram (EEG) recording quality and unavailable clinical hearing scores, leaving a final participant sample of fifteen subjects (9 males, 6 females). The remaining 15 participants were between 36 and 74 years of age (*M* = 59.1, *SD* = 12.0; Table 1). The participant sample varied in terms of device implantation, and included three unilateral, six bimodal (hearing aid and CI), and six bilateral CI users (Table 1). Unilateral and bimodal users were on average 55.2 years old (*SD* = 12.4) on the date of implant activation, while the average age of bilateral CI users on the implant activation date for the ear they used for the study was 50.5 years old (*SD* = 13.5). All methods and protocols used in the current study were approved by the Research Ethics Board at Sunnybrook Heath Sciences Centre (REB #474-2016) in accordance with the Tri-Council Policy Statement: Ethical Conduct for Research Involving Humans. All participants provided written informed consent and received monetary compensation and full reimbursement for parking at the hospital campus for their time and participation.

**TABLE 1:** Demographics of cochlear implant listeners included in the current study.

ID	Age	Sex	Implant side	Ear used	CI use (year)	CI brand	Processor	Strategy	Etiology
CI01	62	F	Right	Right	5	Advanced Bionics	Harmony	HiRes Optima S	Unknown
CI02	36	M	Bilateral	Right	6	MED-EL	SONNET	FS4	Cogan’s Syndrome
CI03*	53	F	Right*	Right	8	MED-EL	OPUS 2	FS4-P	Unknown
CI04*	62	M	Left*	Left	2	Advanced Bionics	Naída Q90	HiRes Optima S	Hereditary
CI05*	74	F	Right*	Right	5	MED-EL	OPUS 2	FS4-P	Hereditary
CI06	63	M	Bilateral	Left	4	Advanced Bionics	Naída Q90	HiRes Optima S	Meniere’s disease
CI07	62	M	Bilateral	Left	2	MED-EL	SONNET	FS4-P	Unknown
CI08	60	M	Bilateral	Left	16	Advanced Bionics	Naída Q70	Unknown	Hereditary
CI09*	44	F	Left*	Left	1	MED-EL	SONNET	FS4-P	Unknown
CI10	71	M	Bilateral	Right	5	MED-EL	OPUS 2	FS4-P	Unknown
CI11	60	F	Bilateral	Right	11	Cochlear	Freedom	ACE	Congenital
CI12	38	F	Left	Left	1	MED-EL	SONNET	FS4-P	Meningitis
CI13	73	M	Left	Left	8	MED-EL	RONDO	FSP	Meniere’s disease
CI14*	72	M	Left*	Left	1	MED-EL	SONNET	FS4-P	Unknown
CI15*	56	M	Right*	Right	6	MED-EL	SONNET	Unknown	Unknown

*Participant used a hearing aid on the non-implanted side in daily life.

### Clinical speech perception scores

Clinical speech perception was evaluated using the AzBio Sentence Test (Spahr et al., 2012). During testing, the 20-sentence lists were presented in quiet, and in noise at an SNR of +5 dB. Testing took place during clinical visits and were administered by audiologists as needed. During testing, participants were seated in a sound-isolated room with minimal reverberation, 1 m away from a loudspeaker positioned directly in front of the participant at approximately the level of a typical listener’s head (Minimum Speech Test Battery (MSTB) For Adult Cochlear Implant Users, 2011). Participants were required to repeat back the sentence heard, and each sentence was scored as the number of words correctly reported. Percent scores are calculated for each sentence based on the percentage of words correct within the presented sentence. Sentence scores were averaged and used as the estimate of speech perception ability within the listening situation. For the current study, the most recent post-implantation test scores relative to the study date for the tested ear of participant (or both ears if individual ear scores were unavailable) were collected (Table 2). The average AzBio scores were 86.9 for listening in quiet (*SD* = 8.51), and 60.2 for listening in noise (*SD* = 26.6).

**TABLE 2:** AzBio performance of CI listeners in quiet and in noise (SNR + 5 dB) for the ear used during listening task.

ID	Listening side	Tested ear	Score in quiet (%)	Score in noise (%)
CI01	Right	Right	88	22
CI02	Right	Right	97	95
CI03*	Right	Right	74	29
CI04*	Left	Left	71	20
CI05*	Right	Both	92	50
CI06	Left	Left	95	56
CI07	Left	Both	80	31
CI08	Left	Left	90	69
CI09*	Left	Left	79	60
CI10	Right	Right	95	95
CI11	Right	Right	83	70
CI12	Left	Left	93	94
CI13	Left	Left	79	49
CI14*	Left	Left	91	74
CI15*	Right	Right	96	89

*Participant used a hearing aid on the non-implanted side in daily life. The side used during the study is listed as Listening Side. Listening scores for both ears are reported for two participants as their scores for individual ears were not available.

### Experimental setup

#### Listening environment

The continuous listening paradigm took place in a sound-attenuated and electrically shielded booth with minimal reverberation. Participants were seated at the center of the booth, surrounded by a circular ring array of eight speakers located at 0°, ± 45°, ± 90°, ± 135°, and 180°. The speaker ring array was raised 1 m from the ground, with each speaker cone located 0.80 m away from the center of the ring. A computer monitor was placed 0.80 m directly in front of the listener’s head, under the speaker located at 0° azimuth. Audio stimuli were played from the loudspeaker at 0° azimuth located directly in front of the listener, while multi-talker background noise was played from surrounding speakers.

#### Audiovisual stimuli

The stimuli used for the paradigm were 15-min audio and video segments of episodes one to four from the first season of *The Office* television show. The show was chosen as it features naturalistic dialogue with little to no music, as well as its use in previous neuroimaging studies (e.g., Byrge et al., 2015; Pantelis et al., 2015). In addition, the opening sequence containing the theme song of the show was removed to minimize non-speech sounds. To investigate cognitive demand in complex listening conditions, segments of the show were presented in conditions that varied in multi-talker babble noise levels. The babble noise soundtrack used for the current study was adapted from the four-talker babble noise used in the Quick Speech in Noise (QuickSIN) test (Killion et al., 2004).

Prior to the start of the paradigm, bilateral and bimodal CI participants were asked to remove the cochlear implant or hearing aid that was contralateral to the ear chosen for testing. In the case of bilateral participants, the ear chosen for testing was the ear that was first implanted with a CI. Participants were asked to pay attention to the television show, attending to the audio and video stimuli presented from the speaker and monitor located directly in front of the participant at the 0° azimuth. While participants attended to the target stimuli, babble noise was simultaneously presented from the seven speakers located at ± 45°, ± 90°, ± 135°, and 180° azimuth. Each episode was presented in blocks of 5-min segments, with each segment varying in background noise level. Audio-video segments were presented at 65 dB SPL, while the babble noise was varied to achieve the desired signal-to-noise ratio (SNR). In the Low-Noise condition, the SNR of the audio-video segment to the background babble noise was + 15 dB. Audio-video segments were presented in the Moderate-Noise and High-Noise conditions at +10 and +5 dB SNR, respectively. Finally, to account for the benefits of visual cues for speech tracking, segments of only audio were presented in the Audio-Only condition at SNR +15 dB, with the video being replaced by a visual crosshair that served as a visual fixation point. Three runs for each condition were performed for a total of 15-min per condition, with the order of runs and conditions randomized for each participant to avoid bias. The presentation of video and audio segments were processed in MATLAB 2009b (The MathWorks, Natick, MA, USA) and controlled by a Tucker Davis Technologies (TDT, Alachua, FL, USA) RX8 Processor. After each episode, participants were asked to answer nine simple open-response questions on the events that occurred in the episode to ensure that they attended to the stimuli, averaging to 79.63% correct (*SD* = 19.53%).

#### Assessment of self-perceived listening effort

In order to measure self-reported listening effort, participants responded to the first section of the NASA-Task Load Index (Hart and Staveland, 1988) after each 5-min run. The NASA-Task Load Index (NASA-TLX) is a multi-scale self-report that rates an individual’s perceived workload for a task (Hart and Staveland, 1988). The dimensions consist of six ordinal subscales: Mental Demand, Physical Demand, Temporal Demand, Performance, Effort, and Frustration. For the current study, the scales for Physical Demand and Temporal demand were removed. The Effort subscale was merged with the Mental Demand subscale in order to align with the concept of listening effort as defined as the mental energy an individual perceives they need to meet external demands (Pichora-Fuller et al., 2016; Peelle, 2018). Specifically, participants responded to the question “How mentally demanding was the task?” on a 21-point scale to gauge cognitive demand, with the endpoints on the left and right being “Low” and “Very High,” respectively. Additionally, subscales assessing the perceived percentage of words understood and the percentage of conversation understood were added.

### Electrophysiological recording

Electroencephalogram data were recorded using a 64-channel antiCHamp Brain Products recording system (Brain Products GmbH, Inc., Munich, Germany) at a sampling rate of 2,000 Hz. EEG caps were fitted onto the participants so that the electrode corresponding to the typical Cz location according to the 10–20 international system was at the vertex of the skull, as determined using the point of intersection between nasion-to-inion and tragus-to-tragus midpoints. The EEG online reference was a separate reference electrode located slightly anterior to the vertex on the midline, while the ground electrode was located on the midline at the midpoint between the nasion and the vertex. Electrodes in proximity to or overlapping the CI magnet and coil on the side of the tested ear were not used; this ranged from 1 to 3 electrodes across all participants.

#### Electroencephalogram preprocessing

Electroencephalogram recordings were imported into EEGLAB (Delorme and Makeig, 2004) for MATLAB 2019a (The MathWorks, Natick, MA, USA) before being filtered with a Finite Impulse Response bandpass filter from 0.3 to 40 Hz. Stimuli audio segments were simultaneously recorded along with the EEG in an auxiliary channel to maximize the temporal alignment of the audio stimuli to the EEG data. The audio soundtrack was then aligned offline to the recorded audio stream by calculating the cross-covariance to find the corresponding starting time point of the soundtrack itself. Speech envelopes were extracted by calculating the absolute value of the Hilbert transformation of the aligned audio soundtrack. The extracted envelope was then downsampled to the EEG recording sampling rate and appended to the EEG data for later analyses. After alignment, the EEG and audio data were both downsampled to 250 Hz and concatenated together across runs and conditions for each participant. Noisy channels and durations of extremely noisy data were removed prior to artifact removal. Independent Component Analysis (ICA) was conducted on the concatenated data to identify stereotypical physiological artifacts (e.g., eye blinks, oculomotor movements, and cardiac activity) and technical artifacts (e.g., electrode pop and line noise). Components containing these artifacts were manually removed based on the visual inspection of component topographies. An average of 6.53 (10.36%; *SD* = 1.13) components were removed across all participants. The recordings were then separated based on study condition for each participant, and the previously removed channels were interpolated using the spherical splines of neighboring channels (Perrin et al., 1989).

#### Cochlear implant artifact suppression

In the presence of auditory stimuli, the electrical stimulation and radio-frequency signals of CIs impart electrical stimulation artifacts into the EEG recording (Wagner et al., 2018). While ICA has been previously used to identify CI artifacts in EEG recordings involving auditory evoked potentials (Gilley et al., 2006; Miller and Zhang, 2014), the nature of CI artifacts makes their reduction more challenging, especially for continuous tasks. In general, ICA separates statistically independent components from the mixed signal. In the case of artifact reduction in MEG and EEG data, temporal ICA separates signal components based on their temporal independence. Artifacts that are not temporally aligned to the stimulus onset (e.g., eye blinks), are therefore separated from sources that are temporally aligned (e.g., event-related potentials). CI artifacts, however, are temporally aligned to the sound onset in a continuous EEG recording, and have a similar morphology to the sound envelope (Mc Laughlin et al., 2013).

Second-order blind identification (SOBI) was applied (Belouchrani et al., 1997) to reduce CI-related artifacts as we have used previously (Paul et al., 2020, 2021). As the name suggests, SOBI is a blind source separation technique that uses second-order statistics in order to separate temporally correlated sources (Belouchrani et al., 1997). SOBI was performed on the continuous EEG data for each participant, and components of suspected CI artifacts were visually identified based on the topographical centroid and implant side used during the listening paradigm. Between 0 and 2 (∼2.51%) components corresponding to the artifact centroids were removed for each participant (*M* = 1.58, *SD* = 0.62). After CI artifact suppression, the cleaned EEG and aligned audio stimuli were subsequently imported into Fieldtrip (Oostenveld et al., 2011) and re-referenced to an average reference before further processing to prepare for temporal response function (TRF) and alpha power analysis.

### Data analysis

#### Temporal response function calculation

Temporal response functions (TRFs) were estimated from EEG data and the speech envelope of the audio signal using the mTRF Toolbox 2.0 (Crosse et al., 2016) in MATLAB. The processed audio signal and the EEG signal were filtered by a 1–20 Hz bandpass, 2nd order, zero-phase filter. The filtered EEG and audio signals were subsequently z-scored for each participant before TRF calculations. Integration window of time lags between −100 and 500 ms were chosen for analysis. The *mTRFcrossval* function was used to optimize the regularization parameter that reduces the overfitting of the speech envelope to the EEG data. The *mTRFcrossval* function performs a leave-one out *N*-fold cross-validation, where for each participant TRFs are estimated for all trials except one. The estimated encoding model is then used to predict the EEG signal for the trial that was omitted, and the Pearson’s correlation coefficient is calculated to assess the correlation between the predicted and actual EEG signals. This process was repeated for regularization parameters ranging from 2^–5^ to 2^15^.

While some studies suggest the use of uniquely optimized regularization parameters for each participant dataset to maximize the TRF estimation (Crosse et al., 2021), this can potentially overfit the TRFs to remnant CI artifacts in CI user recordings. Thus, the correlation coefficients were then averaged across all subjects, channels, and conditions for each regularization parameter, and the value associated with the highest correlation value was chosen as the optimal parameter to use. The continuous EEG data was therefore epoched into 60-s trials prior to TRF calculation, and 2^11^ was selected as the regularization parameter. TRF epochs were averaged within each condition for every participant, generating four sets of TRFs per participant for each listening condition.

#### Neural speech tracking analysis

**FIGURE 1:**
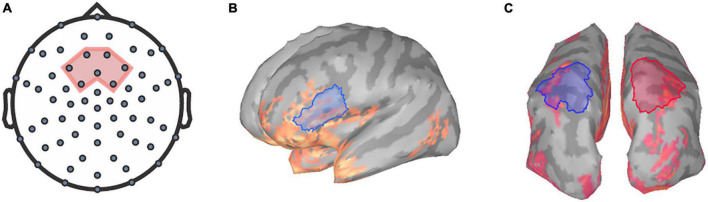
Regions of interest for TRF and frequency analysis. The seven frontocentral channels, EEG channels **(A)** chosen for TRF analysis are within the region shaded in red. Relative alpha power was extracted at the **(B)** left inferior-frontal gyrus and the **(C)** superior-parietal regions for both hemispheres (left in blue, right in red).

Prior to analysis, TRFs for each channel were baseline corrected by subtracting the mean of the values between the baseline period (−100 to −8 ms) from each point in the TRF waveform using Brainstorm (Tadel et al., 2011). Previous literature has identified that speech tracking component amplitudes reaching their maximum magnitudes within the frontocentral region (e.g., Ding and Simon, 2012; Fiedler et al., 2019). Thus, we focused on the frontocentral scalp region as the region of interest (ROI) at the sensor level. Specifically, the TRFs for seven frontocentral EEG sensors within the chosen ROI were averaged for each participant per condition to generate a mean TRF for further analysis (Figure 1A).

To identify the time windows of interest containing the relevant speech components, the babble noise audio was used to create a comparison standard with similar spectral but a temporally different speech envelope. As with the stimuli soundtrack, the absolute value of the Hilbert transformation was calculated for the babble comparison soundtrack to extract the amplitude envelope. The comparison soundtrack was then scaled to the root-mean-squared amplitude of attended soundtrack amplitude before being subjected to the same TRF estimation procedure. TRF differences between the stimuli TRF and the babble noise TRF at each time lag were first compared using two-tailed paired *t*-tests. *T*-test values were then corrected using the Benjamini-Hochberg procedure (Benjamini and Hochberg, 1995). TRF differences at time lags were considered significantly different if the adjusted *p*-values were less than the alpha criterion of 0.05. Component TRF peak amplitudes and peak latencies were then extracted by finding the point of the local maxima/minima of the component within the identified time lag windows.

#### Alpha power

The involvement of the IFG in effortful speech listening has been examined in various studies (e.g., Alain et al., 2018; Price et al., 2019). We have previously shown that neural tracking and alpha power within the left IFG has been observed to predict successful trials for speech perception and be correlated with listening effort (Dimitrijevic et al., 2019). Additionally, previous literature has indicated that parietal alpha oscillations may reflect cross-modal attentional modulation (e.g., Misselhorn et al., 2019). Relative alpha power was extracted from EEG data using Brainstorm following the methods outlined in Niso et al. (2019). The preprocessed spontaneous EEG recordings were first imported into Brainstorm, and source activations were calculated using standardized low-resolution electromagnetic tomography (sLORETA) modeling (Pascual-Marqui, 2002). sLORETA models provide estimates of the power and location of the neural generators that underlie electrophysiological processes. Boundary element model (BEM) head models were created using the OpenMEEG plugin prior to sLORETA modeling. sLORETA models were z-scored, and alpha power was computed using the Brainstorm resting-state pipeline as outlined in Niso et al. (2019).

The power spectral densities (PSDs) of each voxel of the sLORETA models were estimated from 0 to 125 Hz by applying Welch’s method using a window of 1 s at 50% overlap to obtain a 1.0 Hz frequency resolution. Spectrum normalization was then applied by dividing the PSDs by the total power. PSDs were then averaged across all participants and sides for each condition to identify ROIs for alpha analysis. Alpha power was extracted over the left IFG ROI, a 38.74 cm^2^ region encompassing the pars triangularis and the pars opercularis (Figure 1B) as defined by the Desikan-Killiany Atlas (Desikan et al., 2006). Additionally, ROIs encompassing the superior-parietal cortices (Desikan et al., 2006) were also defined as a 57.32 cm^2^ area within the left posterior parietal region and a 57.45 cm^2^ area within the right posterior parietal region (Figure 1C). Mean relative alpha power was calculated by averaging alpha power values between 8 and 12 Hz and then dividing by the total power. The relative alpha power was then extracted over all ROIs for each participant per condition.

### Statistical analysis

All statistical analyses were performed in R (R Core Team, 2021) and MATLAB 2019a (MATLAB, 2019). One-way repeated measures analysis of variance (RM-ANOVA) with the main effect of Condition (Low-Noise, Moderate-Noise, High-Noise, Audio-Only) were used to compare behavioral scores. Two-way factorial RM-ANOVAs with the main effect of Condition and TRF Component (TRF_100_, TRF_200_, TRF_350_) were performed to compare the effect of background noise and visual cues on TRF component amplitudes. Two-way RM-ANOVAs with the main effects of Condition and Listening Side (Ipsilateral to implant, Contralateral to implant) and a paired *t*-test were performed to compare relative alpha power for the superior-parietal and IFG ROIs, respectively. In cases where sphericity is violated, Greenhouse-Geisser corrections were applied to *p*-values (p_*GG*_). For all tests, the alpha criterion was set at 0.05, and *p*-values (p_*adj*_) were adjusted for multiple comparisons using the Benjamini-Hochberg procedure (Benjamini and Hochberg, 1995). Generalized eta squared (η^2^_*G*_) values are also reported alongside ANOVA test statistics to indicate effect size.

Linear mixed models were applied to predict listening demand, perceived percentage of words understood, and perceived percentage of the conversation understood from the fixed effects of TRF component or relative alpha power, listening condition, and subject age using the R package *lme4()* v1.1.29 (Bates et al., 2015). The relationship between listening demand and neural variables were analyzed separately for TRF components and relative alpha power. TRF component amplitudes, alpha power values, and age were treated as continuous variables and z-scored prior to modeling. Although by-subject random effects and condition were included as random intercepts and random slopes respectively to account for the variability explained by the aforementioned factors, random effects are not interpreted due to being jointly unidentifiable as each subject was statistically measured once per condition. Additionally, the by-subject random slope for condition was removed from the perceived percentage of words models due to non-convergence. The correlation between the random intercept for subject and the by-subject random slope for condition was removed for the perceived percentage of conversation models due to convergence issues as well. Fixed effects were subsequently analyzed using ANOVAs with degrees of freedom adjusted by the Satterthwaite method (Luke, 2017), and all *post-hoc* analyses were performed through pairwise comparisons of the estimated marginal means. As there were no AzBio score matching the SNR + 15 and + 10 dB conditions, two-tailed partial Pearson correlations while controlling for age were used to correlate neural measures and AzBio scores. Correlations were controlled for age due to the effects of aging on hearing thresholds. The relationship between perceived percentage of conversation understood and AzBio scores were also explored using a two-tailed partial Spearman correlation while controlling for age. For the partial correlations, the alpha criterion was set at 0.05 and *p*-values (p_*adj*_) were adjusted for multiple comparisons using the Benjamini-Hochberg procedure (Benjamini and Hochberg, 1995).

## Results

### Self-reported listening demand, percentage of words, and conversation understood

Average self-reported mental demand ratings during listening in the audiovisual conditions were 6.78 (*SD* = 2.58) for the Low-Noise (LN) condition, 11.21 (*SD* = 3.20) for the Moderate-Noise (MN) condition, and 15.44 (*SD* = 2.61) for the High-Noise (HN) condition. The average mental demand rating for the Audio-Only (AO) condition was 14.62 (*SD* = 2.63). The ANOVA model returned an effect of condition on mental demand ratings [*F*_(3,42)_ = 40.83, *p* < 0.001, η^2^_*G*_ = 0.620].

**FIGURE 2:**
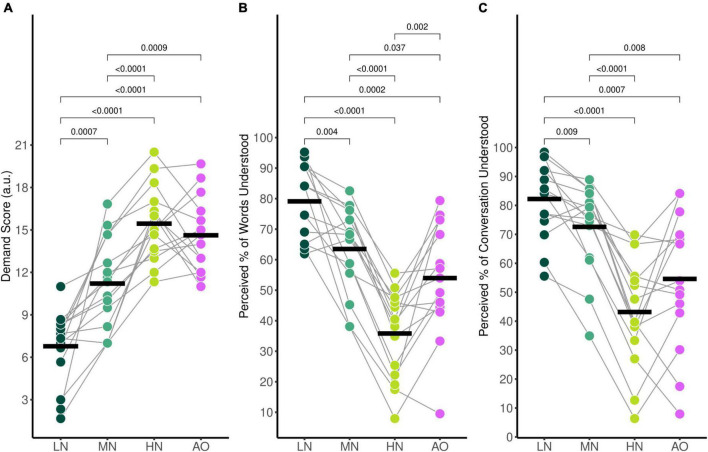
Average self-reported behavioural ratings for **(A)** mental demand, **(B)** perceived percentage of words understood, and **(C)** perceived percentage of the conversation understood. Black horizontal bars indicate the mean score of each condition. Only significant comparisons are displayed.

As expected, self-reported mental demand ratings increased as background babble noise levels increased during the audiovisual listening paradigm (Figure 2A). *Post-hoc* analysis using paired *t*-tests revealed that the average demand rating was significantly higher in the HN condition compared to the MN condition (*p*_*adj*_ < 0.001), and the LN condition (*p*_*adj*_ < 0.001). Additionally, the MN condition demand rating was also statistically significantly higher than for LN (*p*_*adj*_ = 0.007). To compare the effects of visual cues on listening effort, the background babble noise level for the LN and AO conditions were both set at SNR +15 dB. *Post-hoc* analysis indicates that the average demand rating for AO was significantly higher than LN (*p*_*adj*_ < 0.001) despite taking place in the same background noise level. Interestingly, while the AO demand rating was statistically significantly higher compared to the MN rating (*p*_*adj*_ = 0.009) as well, it was not significantly different compared to the HN demand rating (*p*_*adj*_ = 0.322) despite the 10 dB difference in SNR between the two listening conditions.

The average percentage of words understood followed the same trends (Figure 2B); participants reported 79.1% (*SD* = 12.0%) of words understood for the LN condition, 63.5% (*SD* = 14.1%) for the MN condition, 35.8% (*SD* = 14.1%) for the HN condition, and 54.0% (*SD* = 18.5%) for the AO condition. There was a significant effect of condition [*F*_(3,42)_ = 36.67, *p*_*GG*_ < 0.001, η^2^_*G*_ = 0.543], with all conditions significantly differing as shown by *post-hoc* pairwise analysis. The percentage of words understood in the LN condition was significantly higher compared to the MN (*p*_*adj*_ = 0.004). HN (*p*_*adj*_ < 0.001), and AO (*p*_*adj*_ < 0.001) condition. The MN percentage rating was significantly higher than the HN (*p*_*adj*_ < 0.001) and AO (*p*_*adj*_ = 0.037) ratings as well. The percentage of words understood in the HN condition was significantly lower compared to the percentage understood in the AO condition (*p*_*adj*_ = 0.002).

Perceived percentage of conversation understood also decreased parametrically as background noise increased (Figure 2C). Participants reported that they understood an estimated 82.2% (*SD* = 13.1%) of the conversation for the LN condition, 72.6% (*SD* = 15.2%) for the MN condition, 43.2% (*SD* = 18.7%) for the HN condition, and 54.6% (*SD* = 23.1%) for the AO condition. The ANOVA model revealed a significant effect of condition [*F*_(2.10,29.36)_ = 27.65, *p*_*GG*_ < 0.001, η^2^_*G*_ = 0.435]. *Post-hoc* analysis revealed that the perceived percentage of the conversation understood differed across all conditions. The percentage rating in the LN condition was significantly higher compared to the MN (*p*_*adj*_ = 0.009), HN (*p*_*adj*_ < 0.001), and AO conditions (*p*_*adj*_ = 0.007). The rating in the MN condition was also higher than the HN (*p*_*adj*_ < 0.001) and AO condition (*p*_*adj*_ = 0.079). Similar to mental demand ratings, the percentage of conversation understood also did not significantly differ between the HN and AO conditions (*p*_*adj*_ = 0.051).

### Effect of increasing background noise on neural measures

#### Effect of background noise on audiovisual-driven TRF components

**FIGURE 3:**
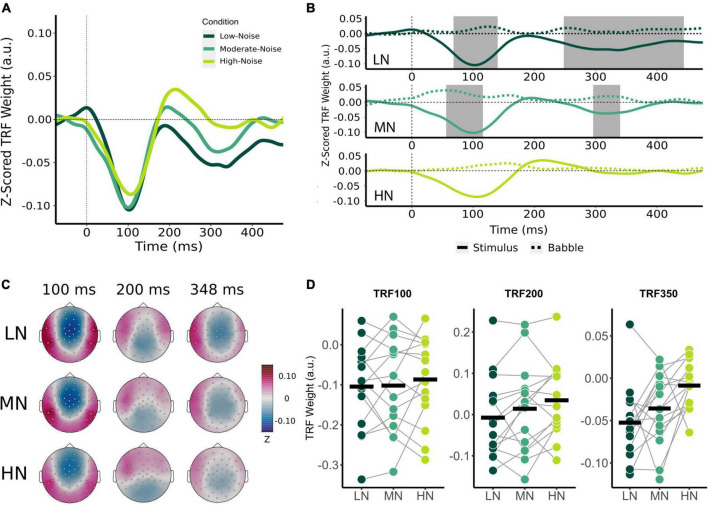
Effect of increasing background noise on sensor-level TRF peaks. **(A)** Mean condition TRFs compared between the audiovisual Low-Noise (LN), Moderate-Noise (MN), and High-Noise (HN) conditions. **(B)** Mean stimuli (solid-line) and background noise (dotted-line) TRFs are compared for each condition (LN—top; MN—middle; HN—bottom) using paired *t*-tests. Shaded regions indicate time intervals where the TRFs were significantly different after correcting for multiple comparisons. **(C)** Topographies of the mean condition stimuli TRFs at the 100, 200, and 350 ms time lags. **(D)** TRF peaks were compared between the LN, MN, and HN conditions. Black horizontal bars indicate the mean TRF weight of each condition. No statistically significant differences between TRF amplitude averages were detected.

Comparison of audiovisual-driven stimuli and babble noise TRFs revealed two significant time lag windows containing negative TRF components for the LN and MN conditions as indicated by the shaded regions in Figure 3B, where stimuli TRFs differed from babble noise TRFs around the 100 ms time lag and the 350 ms time lag (herein referred to as TRF_100_ and TRF_350_ respectively). Visual inspection of the TRF at frontocentral sensors also suggested a TRF component at the 200 ms time lag (TRF_200_; Figures 3A,C), but this did not significantly differ from the babble noise TRFs in each condition. Nonetheless, the TRF_200_ was submitted to statistical analysis to compare across latencies. Based on these responses, an 8 ms time window surrounding the grand mean component peaks were chosen for analysis for the TRF_100_ and TRF_200_. A 48 ms time window surrounding the TRF_350_ component peaks was chosen due to the previously observed wider time lag window width. The average component peak amplitudes were subsequently calculated by averaging the mean stimuli TRF values within the chosen time lag intervals for each participant, and compared between conditions using a two-way RM-ANOVA with the main effect of Condition (Low-Noise, Moderate-Noise, High-Noise) and TRF Component (TRF_100_, TRF_200_, TR_*F*350_).

Initially, mean TRF waveforms of the audiovisual conditions appear to differ in average amplitude for all three speech tracking components (Figures 3A,D). TRF_100_ amplitude seemed to decrease with increasing SNR, with mean amplitudes being −0.104 (*SD* = 0.106) for LN, −0.102 (*SD* = 0.116) for MN, and −0.087 (*SD* = 0.107) for the High-Noise condition. While the mean TRF_200_ component was not significantly different from the babble TRF, TRF_200_ amplitude initially appeared to increase as SNR decreased. The TRF_200_ peak amplitudes were as follows: −0.007 (*SD* = 0.099) for LN, 0.014 (*SD* = 0.108) for MN, and 0.035 (*SD* = 0.090) for HN. TRF_350_ peak amplitudes followed a similar trend to the TRF_100_ component, in that amplitudes decreased as listening condition SNR increased: TRF_350_ peak amplitudes were −0.052 (*SD* = 0.046) for LN, −0.058 (*SD* = 0.051) for MN, and −0.040 (*SD* = 0.032) for HN. While the ANOVA model revealed no significant interaction effect between condition and TRF component [*F*_(2.91,40.75)_ = 0.468, *p*_*GG*_ = 0.701, η^2^_*G*_ = 0.003], the main effect of Condition was statistically significant [*F*_(1.78,24.95)_ = 3.58, *p*_*GG*_ = 0.048, η^2^_*G*_ = 0.027]. *Post-hoc* analysis however revealed no significant differences in general TRF component amplitude between conditions (all *p*_*adj*_ > 0.05).

Beyond the results of the previous analysis, the large variance in TRF amplitudes between participants and the inclusion of the TRF_200_ component (despite the non-significant difference between stimuli and babble-noise TRF_200_ amplitudes) into the omnibus analysis may have lowered its statistical power. Thus, exploratory one-way RM-ANOVAs with the main effect of Condition were conducted separately for each TRF component. The ANOVAs revealed non-significant main effects for the TRF_100_ [*F*_(2,28)_ = 0.347, *p* = 0.710, η^2^_*G*_ = 0.005] and TRF_200_ components [*F*_(2,28)_ = 3.03, *p* = 0.064, η^2^_*G*_ = 0.031]. TRF_350_ component amplitude was found to significantly differ between listening conditions [*F*_(1.84,25.82)_ = 5.47, *p*_*GG*_ = 0.012, η^2^_*G*_ = 0.193]. *Post-hoc* analysis revealed that HN TRF_350_ amplitude was significantly lower than in LN (*p*_*adj*_ = 0.032), but MN TRF_350_ did not significantly differ from LN (*p*_*adj*_ = 0.234) and HN (*p*_*adj*_ = 0.051).

#### Effect of background noise on relative alpha power

**FIGURE 4:**
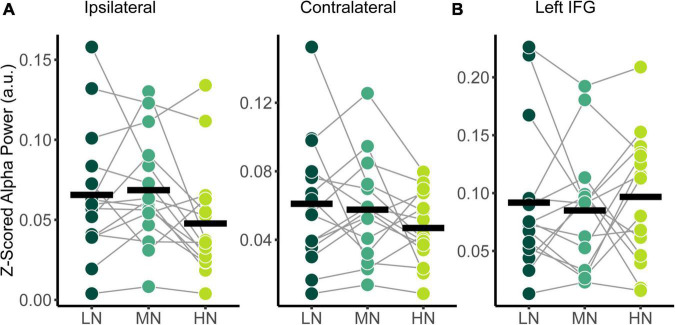
Comparison of relative alpha power between audiovisual Low-Noise (LN), Moderate-Noise (MN), and High-Noise (HN) conditions for the **(A)** ipsilateral and contralateral superior-parietal region and **(B)** left inferior-frontal gyrus (IFG) ROIs. No statistically significant differences were detected.

The power spectral densities for each participant were estimated from the sLORETA models of the spontaneous EEG recordings. Subsequently, the relative alpha power over superior-parietal cortices (Figure 1A) and the left IFG (Figure 1B) were compared between conditions. Relative alpha power at the superior-parietal cortices was analyzed with a two-way RM-ANOVA with the main effects of Condition (Low-Noise, Moderate-Noise, High-Noise) and Listening Side (Ipsilateral, Contralateral), while one-way RM-ANOVA with the main effect of Condition (Low-Noise, Moderate-Noise, High-Noise) was performed for the left IFG. Initially, there appeared to be a parametric increase in relative alpha power at the left IFG as listening condition demand increased. Subsequent analysis, however, revealed no statistically significant interaction effect between Condition and Listening Side for relative alpha power for all ROIs (all *p* > 0.078; Figure 4). Furthermore, the main effect of Condition and Listening Side were also not statistically significant (all *p* > 0.230).

### Effects of the presence of visual cues on neural measures

#### Effect of visual cues on TRF components

**FIGURE 5:**
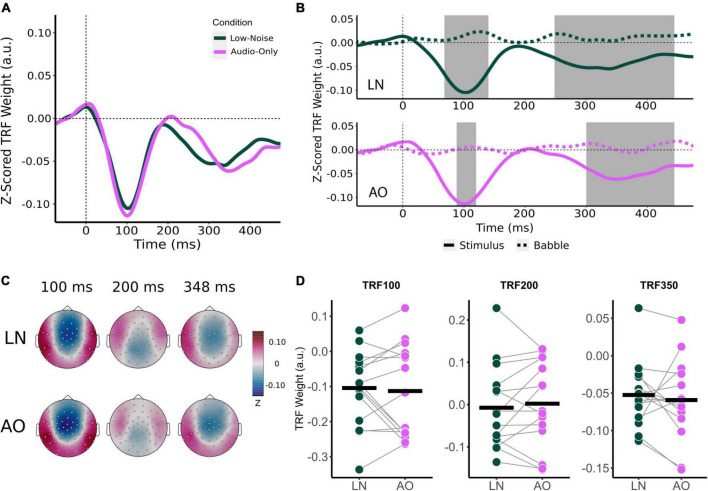
Effect of visual cues on sensor-level TRF peaks. **(A)** Mean condition TRFs compared between the audiovisual Low-Noise (LN) and Audio-Only (AO) conditions. **(B)** Mean stimuli (solid-line) and background noise (dotted-line) TRFs are compared for each condition (LN—top; AO—bottom) using paired *t*-tests. Shaded regions indicate time intervals where the TRFs were significantly different after correcting for multiple comparisons. **(C)** Topographies of the mean condition stimuli TRFs at the 100, 200, and 350 ms time lags. **(D)** TRF peaks were compared between the LN and AO conditions. Black horizontal bars indicate the mean TRF weight of each condition. No significant differences were observed.

Like audiovisual-driven TRFs, the audio-only TRF at the frontocentral censors also contained two significant time lag windows around the 100 and 250 ms time lag (Figures 5B,C). As with the movie TRFs, an 8 ms time window was chosen for analysis for the TRF_100_ and TRF_200_ and a 48 ms time window was chosen for TRF_350_.

Average component peak amplitudes were compared between LN and AO conditions using paired *t*-tests. Like the audiovisual conditions, TRF component amplitudes appeared to change parametrically in the more demanding AO condition (Figure 5D). Compared to LN, which had component amplitudes of −0.104 (*SD* = 0.106) for TRF_100_, −0.007 (*SD* = 0.099) for TRF_200_, and −0.052 (*SD* = 0.046) for TRF_350_, AO TRF components amplitudes were slightly greater. The AO TRF_100_, TRF_200_, and TRF_350_ amplitudes were −0.113 (*SD* = 0.132), 0.002 (*SD* = 0.102), and −0.059 (*SD* = 0.053) respectively. Similar to the analysis for background noise, no significant interaction effect between condition and TRF component was observed [*F*_(2,28)_ = 0.573, *p* = 0.570, η^2^_*G*_ = 0.002]. The main effect of Condition was also not significant [*F*_(1,14)_ = 0.022, *p* = 0.885, η^2^_*G*_ = 0.0001].

#### Effect of visual cues on relative alpha power

**FIGURE 6:**
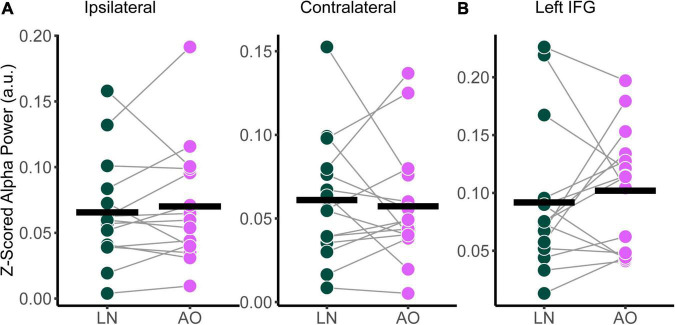
Comparison of relative alpha power between audiovisual Low-Noise (LN) and the Audio-Only (AO) conditions (bottom row) for the **(A)** ipsilateral and contralateral superior-parietal region and **(B)** left inferior-frontal gyrus (IFG) ROIs. No statistically significant differences were detected.

Relative alpha power was analyzed with a two-way RM-ANOVA with the main effects of Condition (Low-Noise, Audio-Only) and Listening Side (Ipsilateral, Contralateral) for the superior-parietal cortices. A paired *t*-test was performed for the left IFG. As with the audiovisual condition comparisons, analyses revealed no statistically significant interaction effect between Condition and Listening Side for relative alpha power the superior-parietal ROI [*F*_(1,14)_ = 1.39, *p* = 0.258, η^2^_*G*_ = 0.003; Figure 6A] and no significant effect of Condition at the left IFG [*t*(14) = −0.674, *p* = 0.512; Figure 6B], despite the apparent parametric change in alpha with condition demand. Furthermore, the main effects of Condition and Listening Side were also not statistically significant for the superior-parietal region (all *p* > 0.218).

### Relationship between neural measures, subjective listening demand, perceived percentage of conversation understood, and AzBio scores

Across all linear mixed models involving TRFs, the Audio-Only condition was set as the reference category to investigate the relationship between changes speech tracking components and behavioral measures as an effect of adding visual cues and increasing background noise levels. The fixed effect of condition was significant across all models (all *p* < 0.001), supporting the previous analysis on differences in subjective behavioral scores between listening conditions. Thus, the fixed effect of condition was not analyzed further owning to the previous analyses performed.

Mixed effects modeling was performed on self-reported listening demand, speech perception during listening, and TRF component amplitude while controlling for participant age. Modeling indicates that demand scores increase as negative-going TRF component magnitudes attenuate and age increases. The analysis reported significant fixed effects of TRF_100_ amplitude [standardized β = 1.961, SE = 0.419, *F*_(1,31.4)_ = 5.461, *p* < 0.001] and age [standardized β = 0.646, SE = 0.267, *F*_(1,7.7719)_ = 5.847, *p* = 0.043]. No other fixed effects aside from condition reached significance (all *p* > 0.110). Regarding the percentage of words understood, no significant fixed effects other than condition was detected (all *p* > 0.069). In contrast, analysis of the mixed model on perceived percentage of conversations understood and TRF component amplitudes while accounting for age also indicates a significant fixed effect of TRF_100_ amplitude [standardized β = −1.418, SE = 1.058, *F*_(1,19.797)_ = 11.361, *p* < 0.001] as well as an interaction effect between condition and TRF_200_ amplitude [*F*_(3,13.071)_ = 3.428, *p* = 0.049]. However, there was no specific interaction effects between TRF components and condition on listening demand (all *p* > 0.090).

**FIGURE 7:**
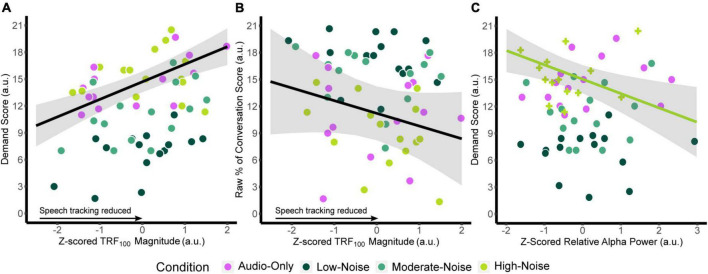
Plots of the function between **(A)** z-scored TRF_100_ amplitude and listening demand ratings, **(B)** z-scored TRF_100_ amplitude and the perceived percentage of conversation understood. The black line indicates the regression line, bounded by the 95% confidence intervals indicated in the shaded gray region, both for visualization purposes. **(C)** Plots z-scored relative alpha power and listening demand ratings in the High-Noise condition. The green line indicates the regression line of the High-Noise condition bounded by the 95% confidence intervals indicated in the shaded gray region for visualization purposes. The plus symbols indicate relative alpha power in the High-Noise condition.

Similar models were fitted for relative alpha power at the superior-parietal and left IFG ROIs. Since there was no significant main effect of Listening Side, the superior-parietal relative alpha power at the ipsilateral and contralateral hemispheres were first averaged for each participant to create a composite measure. ANOVA on the fixed effects revealed a significant interaction between superior-parietal relative alpha power and condition [*F*_(3,13.364)_ = 3.457, *p* = 0.047], specifically in the High-Noise condition (standardized β = −1.992, SE = 0.701, *p* = 0.014). As with the TRF model, the fixed effect of age was also significant [standardized β = 0.6460, SE = 0.267, *F*_(1,7.7719)_ = 5.846, *p* = 0.043]. For the left IFG, ANOVA on the model fixed effects reported no significant terms (all *p* > 0.056). The regression lines and the 95% confidence intervals of z-scored TRF_100_ amplitude against the respective self-report scores are plotted in Figures 7A,B. Weaker TRF_100_ components displaying increasingly positive amplitudes (as TRF_100_ is a negative component) are correlated with higher demand scores (Figure 7A). In tandem, weaker TRF_100_ components are also correlated with a lower perceived percentage of the conversation understood (Figure 7B). Figure 7C shows the mean superior-parietal alpha power is plotted against demand for the High-Noise condition along with the regression line and the 95% confidence interval to show the association between increasing alpha power in the superior-parietal region and decreasing demand ratings.

Two-tailed partial Pearson correlations between TRF component amplitudes and AzBio In-Quiet and In-Noise scores revealed no significant correlations (all *p*_*adj*_ > 0.160). Similarly, no significant correlations were found between relative alpha power and AzBio scores across all ROIs (all *p*_*adj*_ > 0.246). Correlations between AzBio In-Quiet and In-Noise scores and perceived percentage of conversation understood also revealed that while the percentage rating correlated with AzBio In-Quiet and In-Noise scores in the AO condition prior to correcting for multiple comparisons, no correlations survived corrections for multiple comparisons (all *p*_*adj*_ > 0.075).

In summary, TRF_100_ amplitude was correlated to listening demand and proportion of the movie conversation understood and superior-parietal alpha power in the high-noise condition was related to listening demand. No significant correlations were observed for TRF component amplitude and alpha power with clinical AzBio speech perception scores.

## Discussion

The current study investigated neural speech tracking and alpha power and the degree to which these neural measures are reflected in the self-reported mental demand of listening in CI users when attending to naturalistic stimuli. We found: (1) On average, subjects reported higher demand ratings and lower word/conversation understanding as the noise levels increased. Compared to the audiovisual condition, participants presented with audio-only stimuli reported demand and conversation understanding scores that were comparable to the high noise condition, even though the babble noise levels were the same as the low noise condition. (2) Neural tracking/alpha power differences: The neural tracking data showed no significant parametric change in TRF components with increasing babble noise. No differences in neural tracking were observed when the movie visuals were removed. Alpha power did not show parametric changes with noise masker level or with access to visual cues. (3) Relationships to behavior: Neural tracking TRF_100_ was related to self-reported demand and conversations understood, while superior parietal alpha power was only related to self-reported demand.

### Self-reported behavioral scores change concurrently with background babble noise levels, and is offset by the presence of visual cues

In the current study, the Effort subscale of the NASA-TLX was subsumed under the Mental Demand subscale to coincide with the definition of mental effort as “the deliberate allocation of mental resources to overcome obstacles in goal pursuit when carrying out a task” according to the Framework for Understanding Effortful Listening (Pichora-Fuller et al., 2016). In line with previous literature on listening effort and speech intelligibility, self-reported mental demand ratings of CI users attending to audiovisual speech stimuli significantly increased concurrently with background noise levels, supported by similar decreases in perceived percentage of words and conversation understood. Increasing background noise has been shown to require more self-perceived listening effort as indicated by significantly increasing mean NASA-TLX scores of NH listeners attending to conversations and monologs (Peng and Wang, 2019). Accordingly, mean NASA-TLX scores of NH listeners also increases as the spectral resolution of speech (and thereby speech intelligibility) decreased in CI simulations (Pals et al., 2013).

Interestingly, mental demand ratings in the Audio-Only condition with low background noise levels (SNR + 15 dB) were comparable to the demand ratings when attending to audiovisual stimuli in the High-Noise condition (SNR + 5 dB). Additionally, while the perceived percentage of words understood significantly differed between the HN and AO conditions, the percentage of conversation understood did not. When looking specifically at the percentage of words and conversation understood, participants on average reported a ∼20% decrease in understood words in the High-Noise condition compared to the Audio-Only condition. These results indicate that although CI users may perceive fewer words in a SNR + 5 dB listening condition with visual cues, their speech perception and comprehension ability is at a similar level to that of listening without visuals in a lower noise condition. Visual speech cues have been shown to improve the ability of CI users to recognize speech (Dorman et al., 2016). The benefit of visual speech cues on speech intelligibility therefore possibly reduces the mental demand required for CI users to attend to speech in noise. However, prior research on the effect of visual speech cues on subjective listening demand are divergent. For instance, NH individuals reported more difficulty in understanding audiovisual speech stimuli in which the talker wore a non-transparent face mask, compared to when no face mask was worn (Yi et al., 2021). Fraser et al. (2010) observed that NH listeners were more accurate and reported less subjective effort during the audiovisual speech recognition task compared to the audio-only task in equally noisy conditions. In contrast, Brown and Strand (2019) reported that listening effort toward speech in NH listeners did not differ due to the presence of visual facial cues during a dual-task paradigm in hard listening conditions (SNR + 5 dB). Reaction time was instead significantly slower in the audiovisual condition compared to the audio-only condition in easy listening conditions at SNR + 10 dB, indicating that a greater listening effort was required (Brown and Strand, 2019).

The increase in subjective demand in the Audio-Only condition may be due to the degree to which visual speech cues influences speech perception for CI users, compared to NH listeners. CI users have been shown to gravitate toward and rely more on visual speech cues, especially in situations with poor speech intelligibility (e.g., Mastrantuono et al., 2017; Wang et al., 2020). In fact, individuals with hearing loss have been previously observed to be innately biased toward visual speech cues compared to NH listeners (Stropahl et al., 2017). Furthermore, individuals with hearing loss and CI users display cross-modal recruitment of auditory regions by visual stimuli (e.g., Nishimura et al., 1999; Winn et al., 2013; Anderson et al., 2017). Anderson et al. (2017) has shown that the post-operative increase in cross-modal superior temporal cortex activation due to visual speech cues was positively correlated with increases in speech understanding. Thus, while CI users demonstrate greater listening effort compared to NH listeners during everyday listening, it is possible that they also receive greater benefit from visual speech cues in the reduction of listening demand.

### Influence of background noise and visual cues on TRF components

The current study produced speech tracking waveforms with components that have a similar morphology to potentials commonly identified as part of the cortical auditory evoked potential (i.e., N1, P2, N2). Audiovisual-driven TRFs at frontocentral sensors in conditions of increasing background noise resembling N1-P2 cortical auditory evoked potentials (Figure 3A) has been observed in past studies (Hjortkjær et al., 2020; Verschueren et al., 2020). The audio-driven TRF also resemble an N1-P2 cortical auditory evoked potential (Figure 5A). Despite the similarities between the polarity and latencies of the TRF_100_, TRF_200_, and TRF_350_ components to the N100, P200, and N200 event-related potentials respectively, we will not refer to these responses as the classic event-related-potentials due to differences in response characteristics as described in previous literature (Broderick et al., 2018; Reetzke et al., 2021). Recent evidence suggests that TRFs are sensitive to stimulus properties, and are more representative of low-level speech encoding (Broderick et al., 2022; Prinsloo and Lalor, 2022). Accordingly, weaker speech tracking would be indicative of either a deficit in the basic neural processing of speech acoustics, or poor encoding of low-level speech features (Broderick et al., 2022; Prinsloo and Lalor, 2022).

#### General effect of increasing background noise levels on TRF components during audiovisual listening in cochlear implant users

Previous work with NH listeners has shown that background noise and attention both modulate neural speech tracking (Ding and Simon, 2012; Petersen et al., 2017; Verschueren et al., 2020). The magnitude of TRF components were found to decrease as background noise levels increase/SNR decrease (Petersen et al., 2017; Verschueren et al., 2020), even if speech intelligibility remained comparable (Verschueren et al., 2020). Increasing background noise has also been shown to also increase the magnitude of a positive speech tracking component around 200 ms time lag in individuals with hearing loss (Petersen et al., 2017). Concerning the attentional modulation of speech tracking, hearing loss has been shown to negatively affect attentional modulation. Greater degrees of hearing loss were associated with changes in the tracking of the ignored speech stream, leading to smaller differences between the neural tracking of attended and ignored speech streams (Petersen et al., 2017).

Although a general effect of background noise on TRFs was detected, the difference in specific component amplitudes between listening conditions was non-significant. Individual differences between CI users may explain the lack of difference in group-level TRF component amplitudes despite the main effect of increasing background noise, reflecting the large variability in CI outcomes reported in past studies (Blamey et al., 2012; Lazard et al., 2012). Some factors to be considered are the type of CI user (i.e., unilateral, bilateral, bimodal), CI device limitations, and individual differences in effort and attention. The present study contains unilateral, bimodal, and bilateral CI users who may have different subjective listening experiences, and thus different effort and demand requirements. For instance, bilateral CI users have reported a lower degree of listening effort compared to unilateral CI users (Hughes and Galvin, 2013; Schnabl et al., 2015; Kocak Erdem and Ciprut, 2019). The neural response of CI users may also have been limited by individual differences related to the user device, such as CI distortions to the speech envelope, and thus leading to no detectable changes in TRF amplitude at a group level that corresponded with listening effort and attention. Interestingly, TRF_200_ components were not significantly different from the babble noise TRF waveform in all listening conditions in contrast to the TRF_100_ and TRF_350_ components compared to previous studies with NH listeners (e.g., Ding and Simon, 2012; Broderick et al., 2018). This perhaps is another indicator that TRF components represent different aspects of low-level speech encoding, and that specific encoding processes reflected in the TRF_200_ component are impaired in CI users.

It is possible that the effect of background noise on speech tracking is driven by the TRF_350_ component, as exploratory analysis suggested that the late component magnitude was lower in the High-Noise condition compared to the Low-Noise condition. Recently, Paul et al. (2020) reported that bilateral CI users demonstrate stronger speech separation as reflected in later speech tracking differences around 250 ms between attended and ignored speech in a concurrent digit-stream task, while speech tracking toward the attended speech stream was stronger in the early component around 150 ms for NH listeners. Although CI users’ TRFs in the current study demonstrated differentiation of speech from background noise, the later negative component initially appeared to be enhanced for stimuli TRFs, contrary to the stronger late cortical representation of the ignored speech stream described by Paul et al. (2020). Furthermore, the mean late negative component seen in the present study peaked 100 ms later at ∼350 ms, possibly as a result of using continuous speech stimuli instead of number sequences since TRF latency has been linked to speech prosody (Verschueren et al., 2020). Additionally, both TRF_100_ and TRF_350_ were significantly different from the babble TRF for the High-Noise listening condition, suggesting that cortical speech differentiation was intact for low and moderate noise conditions. Speech envelope TRFs have also been shown to remain relatively stable in quiet conditions, despite modulations of speech comprehension via the scrambling the words presented in the speech narrative stimuli (Broderick et al., 2022). Late cortical differentiation of speech may therefore be more sensitive to background noise levels in specific regard to low-level speech encoding, as the TRF_350_ component was not statistically significantly different from the babble noise TRF in the High-Noise condition.

#### Speech tracking components did not differ for audiovisual manipulations in low background noise scenarios

Despite the reported benefits of visual speech cues for speech perception in noise, speech tracking component amplitudes did not differ between the Low-Noise and Audio-Only conditions. The lack of difference is potentially explained by a ceiling effect regarding speech tracking. As the SNR in both audiovisual Low-Noise and Audio-Only condition was relatively high (+15 dB), participants may have been able to track the attended speech stream at the same level of performance for both conditions, despite the increased subjective listening demand in the Audio-Only condition. The benefits of visual cues then present itself in listening situations with lower SNRs, when CI users cannot adequately rely on acoustic speech cues to identify and maintain separate speech streams. However, previous research also suggest that in audiovisual conditions, CI users favor a top-down attentional modulation process when confronted with incongruent visual information, compared to NH listeners that adopt a more bottom-up process (Song et al., 2015). Since the CI users of the current study were not instructed to close their eyes, the irrelevant visual environment in the Audio-Only condition could influence the attentional modulation process by serving as a distractor.

### Early speech tracking component amplitudes are correlated with self-reported demand and percentage of conversation understood, but not the percentage of words understood

The current study found that the strength of early TRF components explained mental demand ratings; more positive (i.e., weaker) TRF_100_ amplitudes are associated with increased perceived listening demand, regardless of background and audiovisual manipulations. Weaker TRF_100_ amplitudes are also correlated with a decreased perceived percentage of conversation understood, but not the perceived percentage of words understood, reflecting recent findings by Broderick et al. (2022) where word recognition remained high in participants despite the scrambling of the speech narrative stimuli. The non-significant relationship observed for TRF_200_ and TRF_350_ components has also been reported in previous literature. Müller et al. (2019) found no significant correlations between listening effort and later component amplitudes in NH listeners after measuring listening effort subjectively through self-reports and objectively using cross-correlations between the speech envelope and EEG signals.

These results suggest that differences in speech encoding between CI users may be strongly reflected in TRF_100_ amplitude. Despite the large variance in CI user characteristics that may have masked the effects of background noise and visual cues on speech tracking at a group level, the relationships between TRF_100_ amplitude, mental demand, and conversation understanding remained statistically significant. TRFs being a visual representative of the quality of low-level speech encoding (Broderick et al., 2022; Prinsloo and Lalor, 2022), can serve as an indicator of deficits related to upstream processes in auditory processing, that is, the relaying of acoustic information from the periphery auditory system to the central auditory system (i.e., downstream) in CI users. Poor encoding of the speech stimuli, whether due to poor encoding of low-level acoustics or deficits in basic auditory processing, would thereby lead to an increase in mental demand when interpreting speech signals, in turn resulting in poor comprehension. Increasing listening demand may also force CI users to compensate by relying on visual speech cues, which may not fully recoup the effects of degradation in auditory information. Alternatively, the concurrent increase in TRF_100_ and demand ratings may represent a potential ceiling effect related to speech intelligibility (e.g., Drennan and Lalor, 2019; Verschueren et al., 2020). Whether due to low background noise or information from visual cues, participants may not have had significant issues understanding the stimuli for the neuromodulatory effects on speech tracking to significantly presents itself in the SNR + 15 dB listening conditions, despite their perception of increased demand and decreased understanding.

The non-significant correlation between AzBio scores and self-perceived percentage of conversation understood is similar to the weak correlation between clinical speech perception tests and subjective QoL outcomes seen in previous studies (e.g., Ramakers et al., 2017; McRackan et al., 2018; Thompson et al., 2020). For instance, Ramakers et al. (2017) reported weak to moderate correlations between Utrecht Sentence Test with Adaptive Randomized Roving sentences performance and the Speech, Spatial, and Qualities of Hearing Scale (SSQ) (Gatehouse and Noble, 2004) Speech scale (*r* = −0.36, *p* = 0.0429) and Nijmegen Cochlear Implant Questionnaire (NCIQ) (Hinderink et al., 2000) Advanced Sound Perception Domain (*r* = −0.47, *p* = 0.0214). Similarly, Thompson et al. (2020) reported that AzBio in noise (SNR 0 dB) scores do not correlate with the Speech-in-Speech Context subscale within the SSQ Speech scale. In turn, a meta-analysis by McRackan et al. (2018) pooled correlation values from studies utilizing tests such as the SSQ and the NCIQ. The pooled correlation values ranged from negligible to low for sentence recognition in quiet (*r* = 0.219 [0.118–0.316]) and in noise (*r* = 0.238 [−0.054 to 0.493]) (McRackan et al., 2018). These results again highlight the complex role of visual cues during listening, as clinical hearing tests are strictly auditory in nature.

### Influence of background noise and visual cues on alpha power

Alpha power is theorized to increase in neural regions involved in processing information irrelevant to the task at hand (Strauß et al., 2014), possibly as a reflection of the inhibition of distracting cortical networks (Jensen and Mazaheri, 2010; Petersen et al., 2015). Changes in alpha power during listening differ depending on cortical regions of interest as well as how the sounds are presented analyzed (i.e., long continuous speech sentences or short duration sounds in event-related paradigms, e.g., see Hauswald et al., 2020). Previous studies have demonstrated that alpha power increases concurrently with task difficulty (Wöstmann et al., 2017), with increases in alpha power at the IFG in tandem with increases in listening effort (Dimitrijevic et al., 2019). Parietal alpha power was also found to increase as listening difficulty and attention increases due to increasing background noise or vocoded speech (Dimitrijevic et al., 2017; Wöstmann et al., 2017). In direct contrast, parietal and frontal alpha power has been shown to decrease as the difficulty of the listening condition increases, whether due to increasing background noise (Seifi Ala et al., 2020), decreasing target stimuli intelligibility (Fiedler et al., 2021), or vocoded speech (Hauswald et al., 2020).

Our results partially conflict with previous research, as alpha power itself at the superior-parietal and left IFG ROIs did not significantly change as an effect of increasing background noise or the presence of visual cues. One interpretation is that the effects of increasing background noise are potentially attenuated by the benefit that visual cues confer for attending to speech, reducing the required resources needed to suppress irrelevant background information. The modulation of parietal alpha power by SNR has been demonstrated for purely auditory tasks (e.g., Dimitrijevic et al., 2017; Wöstmann et al., 2017; Seifi Ala et al., 2020; Paul et al., 2021). The lack of difference in alpha power for the audiovisual listening conditions may therefore be an indicator of audiovisual advantage during sustained listening. At low noise levels in the Audio-Only and Low-Noise condition, background noise affects subjective listening demand, but possibly not enough to warrant neural differences in the suppression of background noise. Superior-parietal alpha power also did not differ between the side ipsilateral and contralateral to the CI side used during the listening task. Increased alpha power has also been reported for the brain hemisphere ipsilateral to the attended stimuli for listening tasks involving spatial attention and suppression of irrelevant stimuli located on the contralateral side (e.g., Foxe and Snyder, 2011; Thorpe et al., 2012; Paul et al., 2020). The target stimuli for the current study were located directly in front of the participants and did not change location. Thus, no lateralization of alpha power was expected. Stimulus material may also influence how alpha power changes as a result of degrading speech clarity. Hauswald et al. (2020) found that when using continuous stimuli parietal alpha power decreased as speech degradation increased, in contrast to studies that used short stimuli such as words or digits (Obleser and Weisz, 2012; Wostmann et al., 2015; Dimitrijevic et al., 2019). The combination of stimulus choice, the benefit of visual cues, and low background noise in some conditions may then have contributed to masking the effect of decreasing alpha power due to decreased speech comprehension across all listening conditions. Alternatively, alpha power as measured in this study may not relate to the conditions of our task design.

Regarding listening demand, a decrease in superior-parietal alpha power was associated with an increase in demand scores, but only in the audiovisual High-Noise condition. Continuous listening in an environment with high background noise would be especially difficult for individuals with hearing difficulty, perhaps to a point where CI users begin to favor visual speech cues over auditory cues to compensates for decreased speech intelligibility. Here, alpha power may represent a release from inhibition (Jensen and Mazaheri, 2010) regarding multisensory integration processes that occur in the parietal region (e.g., Rouger et al., 2008; Misselhorn et al., 2019), similar to the inverted-U pattern displayed for alpha power in high effort listening scenarios (Hauswald et al., 2020; Paul et al., 2021; Ryan et al., 2022). Any association between alpha power and demand scores for conditions with lower background noise may be complicated by CI users’ bias toward visual cues (Stropahl et al., 2017), in that CI users overestimate the benefit that visual cues confer on speech intelligibility, and thereby inherently rely more on visual cues despite the audibility of the speech stimuli. However, it is difficult to determine if this bias is purely subjective based on alpha power alone, due to the cross-modal plasticity observed in individuals with hearing loss (Lazard et al., 2010; Anderson et al., 2017) as well as the aforementioned multisensory integration. Since no visual speech cues were present in the Audio-Only condition, audiovisual integration of visual and auditory speech cues was not necessary, which would lead to the suppression of parietal multisensory integration.

Differences between the effort required for a task (i.e., listening effort) and the difficulty of the task itself (i.e., mental demand) may be reflected in the objective and subjective measures respectively, and thus influencing both TRF components and alpha power. If CI users did not attempt to change their listening effort to match the difficulty/demand of the task throughout all listening conditions, then the differences in both TRF amplitude and alpha power related to listening demand between conditions would be minimized, and thus not detected due to having a small effect size. As the participants were only briefly assessed on stimuli content to gauge attention, passive listening may also have taken place during the task if lapses in attention occurred. Factors beyond attention also influence both speech tracking and listening effort. Even though attentional processes affect listening effort, motivation and fatigue are also confounding factors for both concepts of listening effort and listening demand. Motivation has been found to generally increase subjective listening effort and fatigue when attending to speech in noise, in addition to influencing strategies for coping with effortful tasks (Picou and Ricketts, 2014). Cognitive overload due to task difficulty may also result in participants disengaging from the task itself, thereby producing behavior that is similar to “low effort” (Wu et al., 2016). In addition, familiarity with the stimuli may also play a role in attention, in that familiarity with a voice in a complex listening environment can assist listeners to either attend to or ignore said voice (Johnsrude et al., 2013). When sentences is spoken concurrently by two voices during a word identification task, error rates were found to be higher if both voices were novel, compared to when a familiar voice was the target stimuli or the informational masker (Johnsrude et al., 2013).

## Conclusion

Increasing background noise increased subjective mental demand when listening in noise, while visual speech cues appeared to decrease listening demand in low background noise levels. Stimuli TRF component amplitudes were significantly different from babble noise TRFs (TRF_100_ and TRF_350_ in the low and medium noise conditions) implicating potential difficulties in separating speech from the background for high noise. The significant relationship between early speech tracking components, alpha power, and subjective listening demand suggests that perhaps visual cues play a more complex role for CI users in higher-noise conditions. Relative alpha power however did not appear to change accordingly with background noise in contrast to previous literature. Nevertheless, factors such as study design, motivation, and fatigue may confound the results of neural measures for individuals with CIs compared to NH listeners, and thus warrant further investigation.

Overall, the results of this study suggest that naturalistic multi-talker scenarios such as everyday movie stimuli, can be used to study listening demand in CI users. Neural tracking of continuous naturalistic speech in noise was successfully measured in CI users using TRFs. Meaningful information about the CI user listening experience and the influence of auditory environmental factors can be extracted from brain responses using continuous “ecological” stimuli such as a normal conversation. These findings contribute to the development of naturalistic objective measures of listening effort that may provide more insight into the mental effort and fatigue of CI users in everyday listening. The method of speech tracking estimation used in the current study also does not require an active response from participants, and thus has the potential to be integrated in assessments for individuals that cannot respond to conventional task-based measures. This approach may be especially useful in pediatric populations where behavioral measures of demand and long EEG recordings are difficult to perform.

## Data availability statement

The original contributions presented in this study are included in the article/supplementary material, further inquiries can be directed to the corresponding author.

## Ethics statement

The studies involving human participants were reviewed and approved by the Research Ethics Board at Sunnybrook Health Sciences Centre (REB #474-2016) in accordance with the Tri-Council Policy Statement: Ethical Conduct for Research Involving Humans. The patients/participants provided their written informed consent to participate in this study.

## Author contributions

BX performed data analyses, interpretation, and wrote the manuscript as part of a Master of Science degree thesis that was subsequently adapted for publication. BP and AD contributed to the conceptualization of the work, provided supervision, data analysis, and revised the manuscript before publication. All authors contributed to the article and approved the submitted version.
